# Effect of physical therapy on static postural balance, lower extremity muscle strength and funtional status in children with juvenile idiopathic arthritis

**DOI:** 10.1186/1546-0096-12-S1-P195

**Published:** 2014-09-17

**Authors:** Hristina Colovic, Lidija Dimitirjevic, Ivona Stankovic, Vesna Zivkovic, Marija Spalevic

**Affiliations:** 1 Pediatric Rehabilitation, Clinical Centre of Nis, Nis, Serbia; 2 Clinic Of Physical And Rehabilitation Medicine, Clinical Centre of Nis, Nis, Serbia

## Introduction

Juvenile idiopathic arthritis (JIA) is the most common chronic rheumatologic disease in children. It is defined as permanent arthritis of one or more joints that lasts 6 weeks in children younger than 16 years when all other causes of arthritis are excluded. It is characterized by pain, swelling, morning stiffness in affected joints and decreased quality of life. The goals of physical therapy are to increase joint range of motion, muscle strength, joint stability, physical function without pain and neuromuscular coordination adequate to the age.

## Objectives

The aim of this study was to evaluate the effect of physical therapy on range of motion, muscle strength, balance and functional status in children with JIA.

## Methods

The diagnosis of JIA was made according to the ILAR criteria. Patients with psoriatic arthritis and arthritis associated with enthesitis were exluded from the study.Twenty children (aged 6-18 years) manifesting lower extremity arthritis were assessed. Before and after physical therapy single-leg static balance was measured. Lower extremity strength, active range of motion and functional status (Childhood Healt Assessment Questionnaire - CHAQ) were also assessed at the beginning and the end of the therapy.

Physical therapy included electrotherapy, laser therapy, kinesiotherapy, occupational therapy and hydrotherapy during 3 months. At the beginning all the children had 20 procedures, 5 days in a week. Kinesiotherapy, occupational therapy and hydrotherapy were continued 3 times weekly during 2 months.

## Results

After physical therapy, range of motion, muscle strength, balance and functional status were significantly improved in all the patients ( p< 0.05).

## Conclusion

By increasing joint range of motion and muscle strength, physical therapy improves balance, proprioception and functional status in children with juvenile idiopathic arthritis.

## Disclosure of interest

None declared.

